# Opposing effects of dopamine antagonism in a motor sequence task—tiapride increases cortical excitability and impairs motor learning

**DOI:** 10.3389/fnbeh.2014.00201

**Published:** 2014-06-19

**Authors:** Silke Lissek, Guido S. Vallana, Lara Schlaffke, Melanie Lenz, Hubert R. Dinse, Martin Tegenthoff

**Affiliations:** ^1^Department of Neurology, BG University Hospital Bergmannsheil, Ruhr-University BochumBochum, Germany; ^2^Neural Plasticity Lab, Institute for Neuroinformatics, Ruhr-University BochumBochum, Germany

**Keywords:** motor learning, cortical excitability, fMRI, TMS, dopamine

## Abstract

The dopaminergic system is involved in learning and participates in the modulation of cortical excitability (CE). CE has been suggested as a marker of learning and use-dependent plasticity. However, results from separate studies on either motor CE or motor learning challenge this notion, suggesting opposing effects of dopaminergic modulation upon these parameters: while agonists decrease and antagonists increase CE, motor learning is enhanced by agonists and disturbed by antagonists. To examine whether this discrepancy persists when complex motor learning and motor CE are measured in the same experimental setup, we investigated the effects of dopaminergic (DA) antagonism upon both parameters and upon task-associated brain activation. Our results demonstrate that DA-antagonism has opposing effects upon motor CE and motor sequence learning. Tiapride did not alter baseline CE, but increased CE post training of a complex motor sequence while simultaneously impairing motor learning. Moreover, tiapride reduced activation in several brain regions associated with motor sequence performance, i.e., dorsolateral PFC (dlPFC), supplementary motor area (SMA), Broca's area, cingulate and caudate body. Blood-oxygenation-level-dependent (BOLD) intensity in anterior cingulate and caudate body, but not CE, correlated with performance across groups. In summary, our results do not support a concept of CE as a general marker of motor learning, since they demonstrate that a straightforward relation of increased CE and higher learning success does not apply to all instances of motor learning. At least for complex motor tasks that recruit a network of brain regions outside motor cortex, CE in primary motor cortex is probably no central determinant for learning success.

## Introduction

Motor cortical excitability (CE) has been suggested as a marker of learning and use-dependent plasticity. Primary motor cortex is involved in forming new or adapting existing motor skills (Sanes, 2000). Motor training can increase motor CE (Abbruzzese et al., 1996; Koeneke et al., 2006; Cirillo et al., 2010, 2011), while increased motor CE positively correlates with performance improvements in simple motor tasks (Muellbacher et al., 2001; Ziemann et al., 2001). However, a recent study using a complex motor task found no increase of CE after task training and performance (Lissek et al., 2013), which is in line with two previous studies that used less complex tasks (Ziemann et al., 2001; Smyth et al., 2010). Therefore, it is debatable whether changes in CE in motor cortex can be considered indicators of motor learning and use-dependent plasticity.

The dopaminergic system is involved in both motor learning and the modulation of CE. The ability to learn motor tasks correlates with the level of dopamine (DA) (Garraux et al., 2007) and its metabolites (McEntee et al., 1987). Motor sequencing of single movements requires D2 receptors (Tremblay et al., 2009, 2010). D1 receptors in hippocampus were found involved in associative learning, i.e., in classical conditioning of eyelid responses (Ortiz et al., 2010). In general, motor learning is impaired by DA antagonists and enhanced by DA agonists in animals and humans (Molina-Luna et al., 2009). Dopamine also modulates CE in primary motor cortex: Systemic application of DA antagonists and agonists in humans increased respectively decreased CE in M1 (Ziemann et al., 1996, 1997; Korchounov et al., 2007), suggesting that dopamine enhances inhibition and reduces facilitation in healthy participants (Nitsche et al., 2010).

Thus, the effects of dopaminergic modulation upon CE on the one hand and motor learning on the other appear to work in opposite directions. If DA antagonism increases CE and impairs motor learning, while motor learning alone increases CE, the relation between motor learning and CE with regard to dopaminergic influences remains unclear. To properly examine this relation, the effects of dopaminergic modulation upon CE and motor learning must be measured in the same experimental setup, which has not been done previously.

In our study we used a complex motor sequencing task to investigate the effects of the selective D2/D3 dopamine antagonist tiapride upon CE, motor learning, and the associated brain activation patterns. During a functional magnetic resonance imaging (fMRI) session, participants performed a complex sequence of keypresses under two different conditions. The reproduction condition tested motor learning, while the improvisation condition tapped cognitive flexibility, which appears to be associated with D2 receptor activation (Van Holstein et al., 2011; Stelzel et al., 2013). We hypothesized that overall, detrimental effects of the DA antagonist upon complex motor learning would outweigh its potentially positive effects upon motor CE. In particular, we expected tiapride to enhance motor CE and simultaneously impair learning, reproduction and improvisation of the motor sequence, in parallel to altering activation of brain regions involved in motor learning.

## Materials and methods

### Participants

Thirty two healthy volunteers (20 females, 12 males), mean age: 25.94 years, range: 21–33 years, *SD*: 2.72 years, without a history of neurological disorders participated in this study. All subjects were right-handed as measured by the Edinburgh Handedness Inventory (Oldfield, 1971), with a mean laterality index of +90.31 (*SD*: 9.91, ranging from +63 to +100). Participants received a monetary compensation for their participation (in the amount of €120). Participants were randomly assigned to the experimental (tiapride) and control (placebo) groups. The data of one participant of the tiapride group had to be excluded from the group analysis due to movement artifacts in the fMRI sessions.

### Ethics statement

All subjects participated in this study after giving written informed consent. The protocol was approved by the local ethics committee of the Ruhr-University Bochum. The study conforms to the Code of Ethics of the World Medical Association (Declaration of Helsinki). Prior to the experiments, participants received handouts informing them about the DA antagonist tiapride, the transcranial magnetic stimulation (TMS) and magnetic resonance imaging (MRI) procedures and the instructions for the motor sequencing task.

### Experimental procedure

Prior to administration of the drug, baseline CE was assessed. Then, participants assigned to the experimental group received a single oral dose of 100 mg of the DA-antagonist tiapride, while participants assigned to the control group received an identical-looking placebo. 2 h after administration of the drug/placebo, in accordance with the pharmacokinetic profile of tiapride with peak plasma concentration achieved around this time point (Rey et al., 1982; Norman et al., 1987), the experimental procedure started with the pre-training assessment of CE, followed by training and performance of the motor sequence task in the MRI scanner. Afterwards, CE post-training was assessed. The complete experimental procedure after drug administration took 2–2.5 h, fitting largely into the elimination half-life of tiapride which ranges between 3 and 5 h (Rey et al., 1982; Steele et al., 1993; Dose and Lange, 2000).

#### Motor sequence task

**Figure 1:**
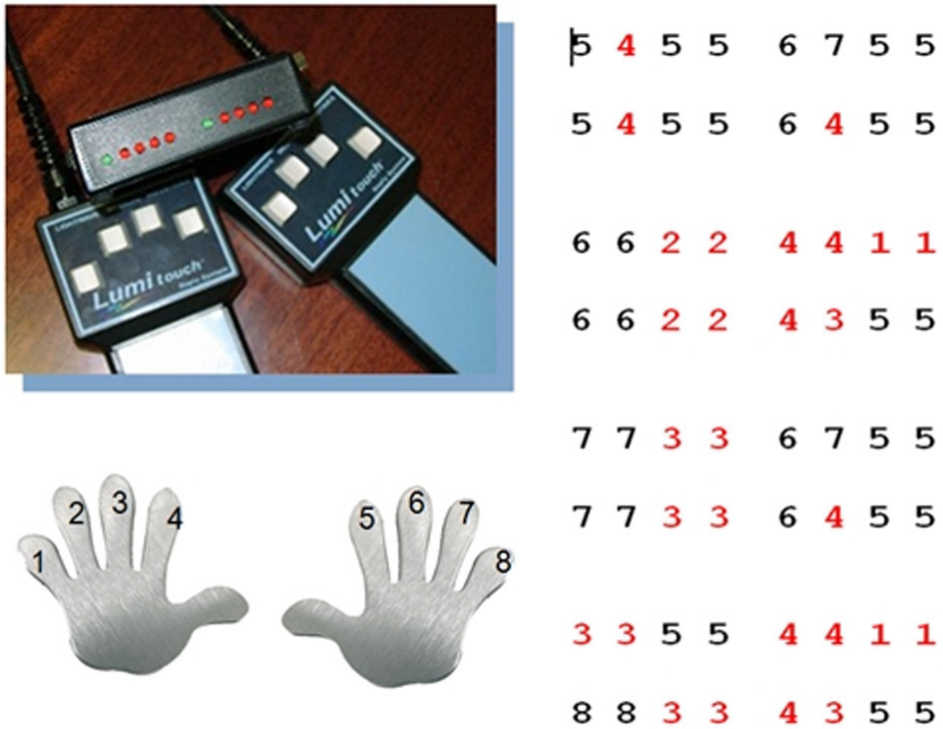
**Motor sequence task**. Participants learned a motor sequence consisting of a series of key presses on two keyboards with each four keys for the left and right hand. Each digit corresponded to a key on the keyboard and thus to a finger, in ascending order from left to right, with 1 corresponding to the left and 8 to the right pinky finger (red digits: left hand, black digits: right hand). The motor sequence consisted of 64 key presses in total, subdivided into four sections with 16 key presses each. In total there were nine different 4 key sequences.

Participants underwent two scanning sessions in the MRT scanner. During the first scanning session participants learned the motor sequence task in an interactive training. They were taught to perform a series of prescribed finger tapping sequences on two fMRI-ready keyboards (LUMItouch response pads, Photon Control Inc., Canada) with four keys each. Each key was associated with a sound of a guitar chord for auditory feedback. In total, there were four motor sequences with 16 key presses each to be learned (requiring 64 key presses in total) (see Figure 1). Each sequence was defined as a series of digits describing the keys and thus the fingers of the left and the right hand in ascending order, excluding the thumbs, with digit 1 corresponding to the left, and 8 corresponding to the right pinky finger. The sequence “4 3 5 5” thus required the following series of key presses: left index finger, left middle finger and two times right index finger. During learning, the prompts and the motor sequences in question were displayed via fMRI-ready LCD-goggles and the sounds were displayed via fMRI-ready headphones (both: Visuastim Digital, Resonance Technology Inc., Northridge, CA, USA). Participants first learned partial sequences with the sequence of keys displayed on the screen, in a second step without visual prompts, requiring participants to play the sequence by rote. Feedback was given regarding the percentage of correct responses, with 80% correct responses defining the sequence as learned. Once the participant could play all single sequences by rote, the process was repeated with the complete motor sequence. Training ended after successful completion of this final part. For participants who did not achieve the training criterion of 80% correct responses, the maximum training duration was set to about 18 min (340 recorded volumes at a TR of 3200 ms). Prior to training in the MRT scanner, participants were instructed to familiarize themselves with the motor sequences on the keyboard, i.e., to memorize the sequences and to train the succession of key presses on life-size photograph printouts of the same keyboards that were later used in the MRT scanner.

The second scanning session evaluated the result of the training session. We used a block design with three conditions: finger tapping as a control condition, a reproduction condition to evaluate correctness of performance of the motor sequence, and an improvisation condition. In the Tapping condition, participants had to alternately press the right and left index finger keys. In the Reproduction condition, participants performed the complete motor sequence acquired during the training. In the Improvisation condition, participants were asked to alter the sequences to their liking while still maintaining a resemblance to the learned sequences. They were also instructed to use all four fingers of both hands during the improvisation. The conditions were presented in alternating blocks, seven per condition. Tapping blocks lasted 25.6 s (8 volumes); Reproduction and Improvisation blocks each lasted 41.6 s (13 volumes), respectively, with each block including an instruction presented for 3.2 s (1 volume), announcing the following condition. The experimental session had a duration of approx. 13 min (238 recorded volumes).

#### Determination of cortical excitability by means of TMS and MEPs

Motor CE was determined three times: the first time before administration of the DA-antagonist to record each participant's baseline excitability, the second time after administration of the DA-antagonist but before the training session in order to check for immediate effects of tiapride upon CE, and the last time after the training session to evaluate the effects of the training in combination with tiapride.

CE was determined by means of single- and double-pulse TMS over primary motor cortex. Magnetic pulses were delivered using a Bistim module connected to two Magstim Rapid 200 (Magstim Co., Whitland, Wales, United Kingdom) stimulators. The stimuli were applied via a flat circular coil (outer diameter 14 cm) centered over the vertex (Cz) with the current flowing counterclockwise in the coil to activate predominantly the left hemisphere, and clockwise to activate predominantly the right hemisphere. This method is a standard procedure for determining CE (Kujirai et al., 1993; Liepert et al., 1997; Schwenkreis et al., 2003). While stimulating the contralateral hemisphere, motor-evoked potentials (MEPs) were recorded by means of Ag-AgCl surface electrodes positioned on the target muscle, the first dorsal interosseus (FDI) muscle of the right hand. The resting motor threshold was defined as the minimum stimulus intensity at which four out of eight stimuli evoked an MEP with an amplitude of >50 μV in the relaxed FDI muscle, expressed in percent of maximum stimulator output. To determine the threshold, stimulus intensity was increased in increments of 5% from a subthreshold level, until threshold and above threshold responses were achieved, then the stimulus intensity was adjusted in order to conform to the above mentioned criteria. Stimulation intensity for single pulses was 120% of the motor threshold thus determined, for double pulses 80 and 120%. A total of six stimulation series was administered, consisting of eight stimulations each: two series of single-pulse stimulation, one before and one after four series of double-pulse stimulations with 2, 10, 4, and 15 ms ISI, respectively. Area and peak-to-peak amplitude of the MEP response were recorded and stored for off-line analysis by means of a NEUROPACK M1 MEB 9200 EVP/EMG measuring system (Nihon Kohden, Japan).

#### Analysis of cortical excitability data

From the data acquired according to the procedure described above, amplitude ratios of CE were calculated by dividing the mean amplitude of the respective double-pulse stimulation series by the mean amplitude of the two sets of single-pulse stimulation series (Kujirai et al., 1993; Schwenkreis et al., 1999, 2003). The ratios were calculated separately for inter-stimulus intervals (ISIs) with inhibitory effects (2 and 4 ms) and ISIs with facilitatory effects (10 and 15 ms). They reflect the degree to which a facilitatory or inhibitory double-pulse stimulation evokes a higher (values > 1) or lower (values < 1) response than the control single-pulse stimulations. Similar ratio methods have also been used in other studies on CE (Ziemann et al., 2001; Smyth et al., 2010). The major advantage of calculating excitability by means of a ratio method instead of reporting raw single or double pulse MEP amplitudes is that this method accounts for potential unspecific changes in overall excitability that might occur between time points of measurement, and thus should deliver more reliable information about changes in facilitation and inhibition.

To check for an overall effect across groups and measuring points upon CE, we first performed a 3 × 2 repeated measures ANOVA with the complete data. Subsequently, within each of the two treatment groups, we performed a One-Way (3 × 1) repeated measures ANOVA with time of measurement (baseline, pre and post) as independent variable. To further evaluate whether administration of the DA-antagonist alone will already affect CE, we used a *t*-test for paired samples to compare baseline excitability with excitability 2 h after administration of tiapride, but prior to the motor training. Moreover, in order to test our main change hypothesis assuming an increase of CE in tiapride after motor training, we compared CE pre and post training by means of a *t*-test for paired samples.

#### Imaging data acquisition

Images were acquired using a whole-body 3T scanner (Philips Achieva 3.0 T X-Series, Philips, The Netherlands) with a 32-channel SENSE head coil. Blood-oxygen level dependent (BOLD) contrast images were obtained with a dynamic T2^*^ weighted gradient echo EPI sequence using SENSE (TR 3200 ms, TE 35 ms, field of view 224 mm, slice thickness 3.0 mm, voxel size 2.0 × 2.0 × 3.0 mm). We acquired 45 transaxial slices parallel to the anterior commissure—posterior commissure (AC-PC) line which covered the whole brain. Two imaging sessions were conducted, the first (maximum 340 volumes) for training of the motor sequence, the second (238 volumes) for the actual experimental task. Additionally, anatomical images of each subject were acquired using an isotropic T1 TFE sequence (field of view 240 mm, slice thickness 1.0 mm, voxel size 1 × 1 × 1 mm) with 220 transversally oriented slices covering the whole brain.

#### Imaging data analysis

For preprocessing and statistical analysis of the fMRI data, we used the Statistical Parametric Mapping (SPM) Software, Version 8 (Wellcome Department of Cognitive Neurology, London, UK) implemented in Matlab (Mathworks, Sherbon, MA). The first three images of each fMRI session (total 340 resp. 238 images), during which the BOLD signal reaches steady state, were discarded from further analysis to remove non-steady state effects caused by T1 saturation. To correct for between-scan movements, all volumes were realigned to the first volume. Functional images were spatially normalized into standard stereotactic coordinates at 2 × 2 × 2 mm^3^ using an EPI template provided by the Montreal Neurological Institute (MNI). Smoothing was conducted with a 6 mm full-width half-maximum (FWHM) kernel, in accordance with the standard SPM procedure. The acceptable limit for head motion was 2 mm for translational movements and 0.5° for rotational movements.

In a first-level single-subject analysis, changes in the BOLD response for each participant were assessed by linear combinations of the estimated GLM parameters which are displayed by the individual contrast images. This analysis was performed by modeling the Reproduction and Improvisation conditions as explanatory variables convolved with a standard hemodynamic response function as implemented in SPM 8. Contrast images were calculated that compared activation between the conditions of Improvisation, Reproduction and the rest condition of Tapping.

In a second-level random-effects analysis, the single subject contrast images were entered into one-sample *t*-tests for each group to display the commonly activated regions for the contrasts Improvisation > Reproduction and Reproduction > Improvisation. In addition, we performed two-sample tests to directly determine areas of differential activation between the two groups. Since the computed contrasts between Improvisation and Reproduction were very close in terms of processing requirements, i.e., the expected effects were rather small, no correction for multiple comparisons was carried out. The resulting statistical parametrical maps were thresholded at *p* < 0.001 uncorrected on the voxel level and a minimum cluster size of ten contiguous active voxels. Nevertheless, because uncorrected statistics were considered imaging results should be interpreted with caution.

In another second-level analysis, we contrasted activation during Improvisation and Reproduction with activation during the baseline condition of Tapping to identify activation differences in motor cortical regions. For the analyses of differential activation in the motor cortex between groups we used an anatomical mask comprising bilateral Brodmann areas 4 and 6 constructed using the WFU Pickatlas (Maldjian et al., 2003). Two-sample *t*-tests comparing tiapride and placebo groups were thresholded at *p* < 0.05 FWE-corrected on the voxel level and a minimum cluster size of *k* = 10.

To evaluate potential relations between activated brain regions and performance in the motor task, or CE, respectively, we extracted mean signal intensities for each participant using the Marseille Region of Interest Toolbox for SPM MarsBaR (Brett et al., 2002) for the clusters found activated during Reproduction and Reproduction + Improvisation in the above mentioned contrasts. We correlated these activation values with the percentage of correct responses, or CE values, respectively. Statistical analyses were performed using the SPSS 20 software package (IBM SPSS Statistics for Windows, Version 20.0, Armon, NY: IBM Corp.).

#### Behavioral data acquisition

Each keypress during the three conditions was recorded to log files in a format that could be read out in statistics and spreadsheet software.

#### Behavioral data analysis

Behavioral data were analyzed regarding correctness in the Reproduction condition and regarding the level of improvisation in the Improvisation condition. The percentage of correct keystrokes was determined by comparing the participant's motor sequence with the prescribed motor sequence. Speed was not taken into account, i.e., if a participant did not complete the motor sequence within one block, his partial sequence was compared to the prescribed sequence of the same length. In addition, we analyzed motor speed in the reproduction condition by calculating the average time between individual keystrokes for each reproduction block and then calculating the mean value over all blocks for each participant. Deviations from the prescribed motor sequence were considered errors. However, the evaluation procedure took into account errors that were obviously corrected after maximal four keystrokes by continuing with the correct sequence.

The level of improvisation in the Improvisation condition was determined by computing the Shannon Entropy H (Shannon, 1948)—a measure of the randomness of a probability distribution of values which is often used to measure diversity in categorical data. The Shannon Entropy H was calculated as the average information contained in the series of improvisations. This calculation of H was performed for each participant based on the amount of preserved strings of minimum four key presses that still corresponded to the prescribed motor sequence, irrespective of their position in the improvised sequence.

## Results

### Cortical excitability

In a first step of analyzing CE data, we calculated a 3 × 2 ANOVA with repeated measures to explore overall effects regarding measuring points and groups. Results demonstrated a significant main effect of time of measurement for both facilitatory [*F*_(2, 29)_ = 3.477; *p* = 0.037] and inhibitory [*F*_(2, 29)_ = 3.391; *p* = 0.040] CE, indicating a change of CE between measuring points across groups. Results for the main effect of group for facilitatory [*F*_(1, 29)_ = 1.058; *p* = 0.312] and inhibitory [*F*_(1, 29)_ = 0.182; *p* = 0.673] CE indicated that the overall CE level did not differ significantly between groups, suggesting that tiapride did not cause a drug-related increase of CE relative to placebo. The time^*^group interaction was significant [*F*_(2, 29)_ = 3.465; *p* = 0.038] for inhibitory CE, but not for facilitatory CE [*F*_(2, 29)_ = 0.492; *p* = 0.614].

In a second step, in order to evaluate our hypothesis of a change in CE within the tiapride group, and to further explore the significant differences between measuring points across groups, we performed additional repeated measures ANOVAs without a group variable.

A One-Way (3 × 1) repeated measures ANOVA showed a significant main effect of time of measurement upon CE in the tiapride group, both for facilitatory CE [*F*_(2, 15)_ = 3.761; *p* = 0.036] and for inhibitory CE [*F*_(2, 15)_ = 5.125; *p* = 0.013]. Mere Administration of the DA-antagonist tiapride did not affect CE—we observed no significant difference between the baseline CE level and the level after administration of tiapride before the training [*t*-test for matched samples; facilitatory CE: *t*_(14)_ = 0.467; *p* = 0.648; inhibitory CE: *t*_(14)_ = 0.923; *p* = 0.372]. In contrast, as hypothesized, the CE level post training differed significantly from pre training in the tiapride group, both for facilitatory CE [*t*-test for matched samples; *t*_(14)_ = 2.511; *p* = 0.0125 one-tailed] and inhibitory CE [*t*_(14)_ = 2.235; *p* = 0.021 one-tailed], reflecting an overall increase in CE after training, i.e., an increase in facilitation combined with a decrease of inhibition.

**Figure 2:**
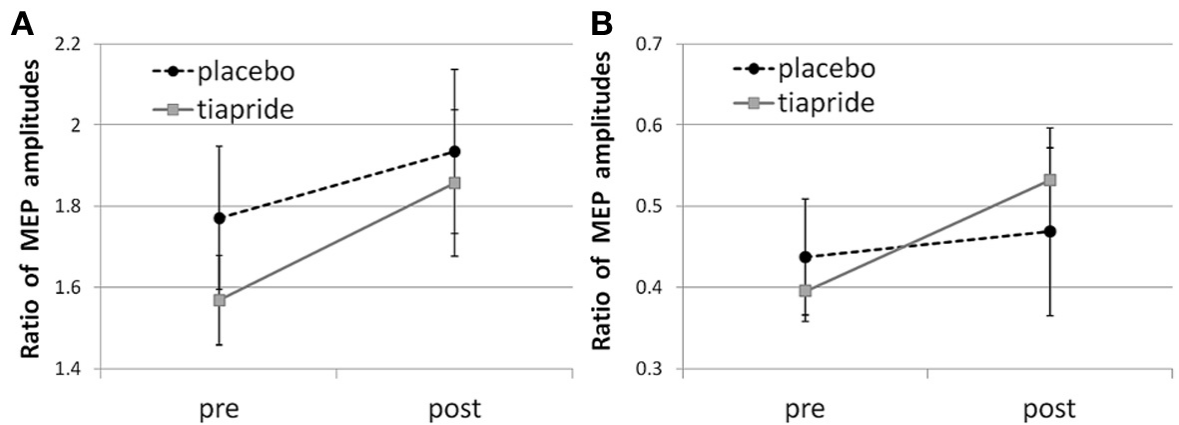
**Cortical excitability in the tiapride and placebo groups before and after learning and training of the motor sequence task**. Mean CE is expressed as the ratio of MEP amplitudes in response to **(A)** facilitatory double pulse/single pulse stimulation and **(B)** inhibitory double pulse/single pulse stimulation pre and post training.

In the placebo group, time of measurement had no significant effect, neither upon facilitatory CE [*F*_(2, 16)_ = 0.666; *p* = 0.521] nor upon inhibitory CE [*F*_(2, 16)_ = 0.685; *p* = 0.512], indicating that neither the administration of placebo nor the motor training altered their CE level (see Figures 2A,B).

Baseline CE levels did not differ significantly between groups [facilitatory CE: *t*_(29)_ = 1.826; *p* = 0.098; inhibitory CE: *t*_(29)_ = 1.202; *p* = 0.239].

Table 1 gives an overview over the CE ratios of the tiapride and placebo groups for the three points of measurement.

**Table 1:** **Cortical excitability baseline, pre and post training in tiapride, and control participants**.

**Measurement**	**CE ratio (±s.e.m.)**	
	**Tiapride**	**Placebo**
Facilitatory CE baseline	1.5113 (±0.0972)	1.7924 (±0.1179)
Facilitatory CE pre	1.5685 (±0.1098)	1.7709 (±0.1760)
Facilitatory CE post	1.8569 (±0.1801)	1.9350 (±0.2018)
Inhibitory CE baseline	0.3522 (±0.0486)	0.5037 (±0.1131)
Inhibitory CE pre	0.3953 (±0.0375)	0.4374 (±0.0714)
Inhibitory CE post	0.5320 (±0.0638)	0.4689 (±0.1034)

#### Cortical excitability itemized according to ISIs

In order to evaluate which of the ISIs we used to measure facilitatory and inhibitory CE accounted for the effect we observed in the tiapride group, we repeated the above analyses for the individual ISIs (2 and 4 ms inhibitory, 10 and 15 ms facilitatory).

In the tiapride group, the One-Way (3 × 1) repeated measures ANOVA showed a significant main effect of time of measurement upon CE for ISI = 4 ms [*F*_(2, 15)_ = 7.926; *p* = 0.002] and ISI = 15 ms [*F*_(2, 15)_ = 3.665; *p* = 0.039]. In both cases, this effect was due to an increase in CE post relative to pre training [ISI = 4 ms; *t*_(14)_ = 2.481; *p* = 0.026 one-tailed; ISI = 15 ms; *t*_(14)_ = 2.756; *p* = 0.015 one-tailed], while there was no significant change from baseline to pre training [ISI = 4 ms; *t*_(14)_ = 1.312; *p* = 0.210; ISI = 15 ms; *t*_(14)_ = 0.785; *p* = 0.446]. For ISI = 2 ms [*F*_(2, 15)_ = 0.589; *p* = 0.562] and ISI = 10 ms [*F*_(2, 15)_ = 1.947; *p* = 0.161] there was no significant effect of time of measurement. Thus, the increased facilitatory CE post training rested predominantly on ISI = 15 ms, the increase in inhibitory CE on ISI = 4 ms.

In the placebo group, time of measurement had a significant main effect only in ISI = 2 ms [*F*_(2, 16)_ = 3.351; *p* = 0.042], which was due to a change pre training relative to baseline [*t*_(15)_ = 2.212; *p* = 0.043], while there was no significant change post relative to pre training [*t*_(15)_ = 1.678; *p* = 0.114]. In all other ISIs, there was no significant main effect of time of measurement [ISI = 4 ms; *F*_(2, 16)_ = 0.006; *p* = 0.994; ISI = 10 ms; *F*_(2, 16)_ = 0.807; *p* = 0.456; ISI = 15 ms; *F*_(2, 16)_ = 0.119; *p* = 0.888].

### Behavioral results

#### Learning duration

The tiapride group spent significantly more time in the training phase (836.27 s ± 72.72 s.e.m.) than placebo (625.2 s ± 79.68 s.e.m.) (*t*-test for independent samples: [*t*_(27)_ = 1.948; *p* = 0.030 one-tailed]. Seven out of 15 tiapride participants did not complete the training within the designated time frame, compared to 4 out of 16 placebo participants.

#### Correctness of performance

**Figure 3:**
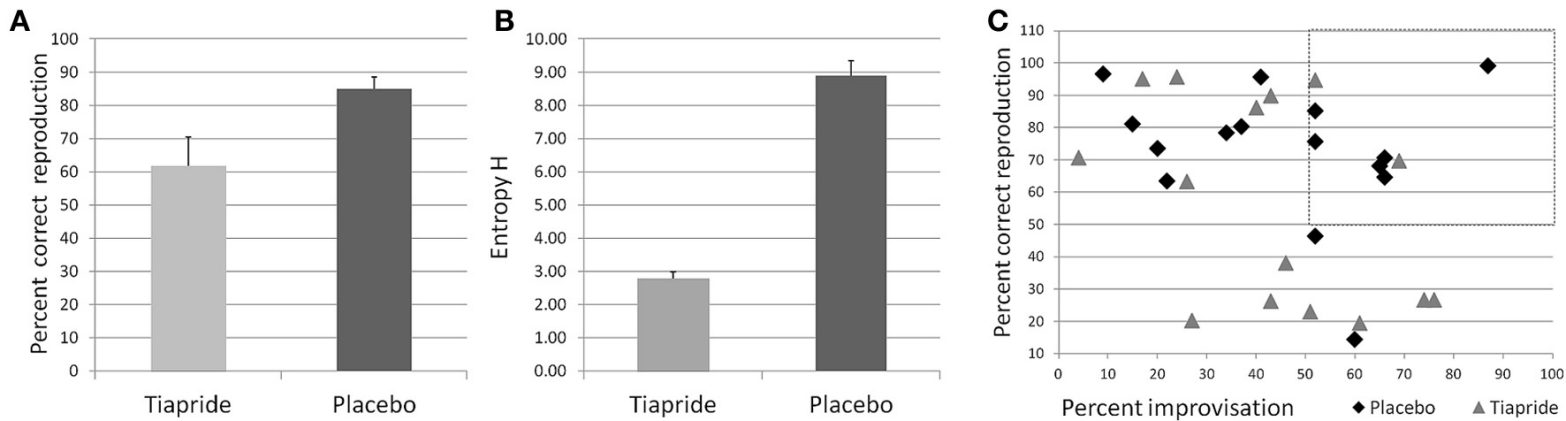
**Performance in the motor sequence task. (A)** Percent correct keypresses in reproduction of the learned motor sequence. **(B)** Total Entropy H produced by the groups during improvisation over the motor sequence. Higher values indicate a higher level of improvisation. **(C)** Scatterplot of the relation between reproduction (x) and improvisation (y) performance in the placebo (diamond) and tiapride (triangle) group. The dashed line rectangle frames the data points of those six placebo and two tiapride participants with both good reproduction and good improvisation (>50%).

Performance in the Reproduction condition was significantly impaired in the tiapride group compared to the placebo group [*t*_(29)_ = 2.393; *p* = 0.024], with a mean percentage of correct responses of 61.75% (±8.638 s.e.m.) in the tiapride group and 84.85% (±3.735 s.e.m.) in the placebo group (see Figure 3A).

#### Speed in reproduction

Motor speed during reproduction of the trained sequence did not differ between groups [*t*_(28)_ = 1.262; *p* = 0.217 two-tailed]. Mean time between keystrokes was 0.7604 s ± 0.03527 s.e.m. in the placebo group and 0.7006 s ± 0.03154 s.e.m. in the tiapride group. Thus, there was no tendency in the tiapride group to favor speed over accuracy which might have contributed to their inferior reproduction performance.

#### Level of improvisation

Regarding mean values over the complete group, there were no significant differences in the level of improvisation in terms of entropy H between the groups [mean placebo H = 1.4817 (±0.07618 s.e.m.); mean tiapride H = 1.3950 (±0.09500 s.e.m.), *t*_(6)_ = 0.593; *p* = 0.575)].

However, actual improvisation over a learned sequence (as opposed to stochastic keystrokes) is possible only if the sequence has been properly memorized, which was not the case in a number of tiapride participants. Significantly more placebo than tiapride participants reached a level of above 50% correct reproduction (placebo: 14 out of 15 participants, tiapride 8 out of 15 participants, χ^2^ = 6.136; *p* = 0.013 two-tailed). For the comparison of improvisation levels we considered only those participants who reproduced correctly more than 50% of the motor sequence and actually showed improvisation, i.e., improvised over more than 50% of the original sequence. In order to account for the higher number of participants in the placebo group than in the tiapride group showing improvisations, we calculated the total entropy H for the participants. As a group, placebo showed a higher entropy value than tiapride (H total of placebo group = 8.88 ± 0.457 s.e.m.; H total of tiapride group = 2.79 ± 0.190 s.e.m.; χ^2^ = 8.0; *p* = 0.046 two-tailed). Figure 3B shows the total entropy H produced by the two groups during the improvisation blocks.

Comparisons of reproduction and improvisation performance showed that significantly more placebo than tiapride participants with good reproduction performance exhibited a higher improvisation level (placebo: 6 out of 14 participants, tiapride: 2 out of 8 participants, χ^2^ = 2.727; *p* = 0.049 one-tailed). In contrast, tiapride participants with good reproduction performance tended to reproduce this sequence also in the improvisation condition (see Figure 3C).

#### Correlation of motor performance and cortical excitability

To evaluate a potential relation between excitability and motor performance, we calculated Pearson's correlation coefficient between CE and percent correct responses. There were no significant correlations, neither in the complete sample nor in the tiapride and placebo subgroups (see Table 2).

**Table 2:** **Correlation between motor task performance and cortical excitability (Pearson's correlation coefficient)**.

	**Tiapride Pearson's *r***	***p* 2-tailed**	**Placebo Pearson's *r***	***p* 2-tailed**	**All Pearson's *r***	***p* 2-tailed**
Facilitatory CE pre training	−0.226	0.419	0.386	0.368	0.135	0.479
Facilitatory CE post training	0.012	0.965	0.454	0.089	0.214	0.257
Facilitatory CE pre + post tr.	−0.082	0.772	0.460	0.084	0.196	0.300
Inhibitory CE pre training	−0.091	0.746	0.278	0.316	0.081	0.669
Inhibitory CE post training	0.194	0.489	0.159	0.132	0.109	0.566
Inhibitory CE pre + post	0.192	0.493	0.158	0.573	0.104	0.583

### Imaging

#### Activation in motor cortex

**Figure 4:**
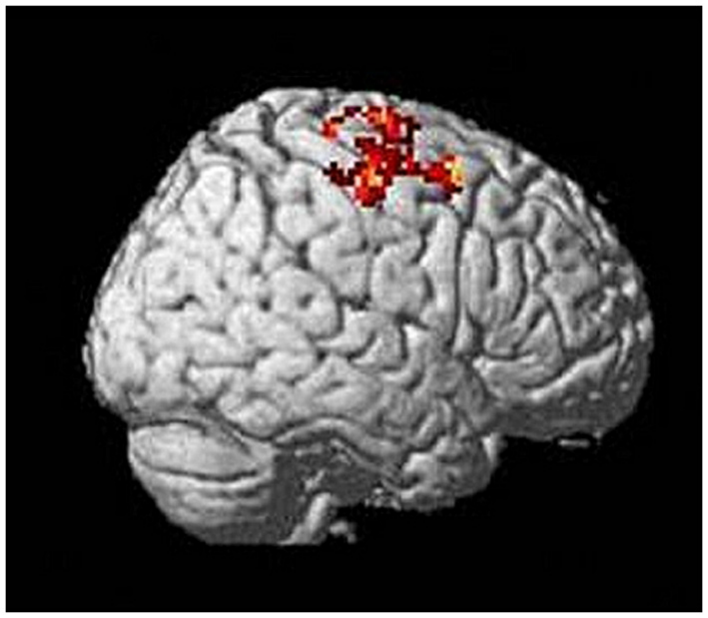
**Higher activation in motor cortex (righthemispheric BA 4 and 6) in the tiapride group during task performance compared to tapping (two-sample *t*-test tiapride > placebo, *p* < 0.05 FWE corrected)**.

To analyze activation in motor cortical areas during task performance, we compared tiapride and placebo groups directly (two-sample *t*-test, contrast reproduction + improvisation > tapping, thresholded at *p* < 0.05 FWE-corrected, *k* = 10). During motor sequence reproduction and improvisation, compared to the control condition, tiapride showed higher activation than placebo in righthemispheric BA 4 and BA 6 (see Figure 4 and Table 3). There was no region in which the placebo group showed higher activation than the tiapride group.

**Table 3:** **Areas in motor cortex that show higher activation in the tiapride group during task performance and improvisation (two-sample *t*-test tiapride > placebo, reproduction + improvisation > tapping, thresholded at *p* < 0.05 FWE-corrected)**.

**Brain region**	**BA**	**Hem**.	**MNI coordinates**	***t*-value**	**Voxel in cluster**
Middle frontal gyrus	6	R	42, 10, 58	5.11	68
Precentral gyrus	4	R	40, −20, 50	4.31	178

***Correlation of motor cortex activation with performance and cortical excitability***. To evaluate potential correlations between BOLD activation in motor cortex and CE as well as performance in the motor task, we calculated mean signal intensities of the activated motor cortical regions. There were no significant correlations of motor performance during reproduction with motor cortex signal intensity, neither in the tiapride nor in the placebo subgroup. For the complete group, we found a significant negative correlation of activation in right-hemispheric BA 6 (MNI coordinates 42, 10, 58) with performance, with higher activation in this region associated with inferior performance (Pearson's *r* = 0.473; *p* = 0.020 two-tailed). Between CE and BOLD activation in motor cortex we found no significant correlation.

#### Activation in task-related brain regions

The two groups activated a largely similar network of brain regions. In improvisation compared to reproduction of the motor sequence, the placebo group showed higher activation in bilateral orbitofrontal cortex (BA 47, 11), bilateral dorsolateral PFC (BA 8, 9, 10), and bilateral superior, middle, and inferior temporal gyrus (BA 21, 20, 22, 38) and inferior parietal lobule (BA 40), moreover regions in right medial frontal gyrus (BA 9, 10) and left anterior cingulate (BA 32). In the same contrast, the tiapride group activated similar brain regions, with higher activation in bilateral orbitofrontal cortex (BA 47, 11), bilateral dorsolateral PFC (BA 8), left superior temporal gyrus (BA 22, 38), bilateral medial frontal gyrus (BA 8, 9, 10) and bilateral anterior cingulate (BA 24, 32). During motor sequence reproduction, compared to improvisation, the placebo group showed higher activation in bilateral precuneus and superior parietal lobule (BA 7, 31), in bilateral cingulate gyrus (BA 24, 32), and in primary motor and somatosensory regions (BA 4, 6, 3). In the same contrast, the tiapride group displayed higher activation in bilateral precuneus (BA 31), bilateral cingulate gyrus (BA 24, 31, 32) and left anterior cingulate (BA 32), in bilateral caudate body and tail, and in a region in right orbitofrontal cortex (BA 11) (see Table 4).

**Table 4:** **Activation in improvisation and reproduction of the motor sequence task in the placebo and tiapride groups (one sample *t*-tests, *p* < 0.001, *k* = 10)**.

**Brain region**	**BA**		**Improvisation > reproduction**						**Reproduction > improvisation**					
			**Placebo**			**Tiapride**			**Placebo**			**Tiapride**		
			**MNI**	***t*-value**	**Voxel**	**MNI**	***t*-value**	**Voxel**	**MNI**	***t*-value**	**Voxel**	**MNI**	***t*-value**	**voxel**
**FRONTAL CORTEX**														
Precentral gyrus	4	R							14, −24, 52	5.10	82			
Medial frontal gyrus	6	R							12, −30, 56	5.88	82			
	8	R				0, 42, 44	5.04	44	10, 16, 52	4.47	33			
	9	R	4, 52, 40	5.40	1338	0, 40, 36	5.60	44						
	10	L	−6, 54, 10	4.33	93	−4, 60, 18	5.56	22						
		R	2, 54, −4	3.76	49									
Inferior frontal gyrus	9	L							−52, 4, 30	5.63	552			
	47	L	−46, 34, −12	9.07	86	−28, 26, −18	7.98	557						
						−48, 22, −8	7.60	557						
						−24, 14, −22	6.33	57						
		R	60, 18, −4	5.39	44	52, 36, −10	7.05	325						
						38, 32, −20	6.13	325						
	45	R				64, 16, 6	5.43	13						
Middle frontal gyrus	6	R							26, 2, 48	4.20	12			
	47	L	−48, 35, 0	4.85	747									
		R	48, 40, −4	3.94	298									
	11	L	−34, 35, −14	4.42	747	−44, 44, −16	6.16	91						
		R										28, 40, −6	4.93	22
												20, 40, −16	3.92	16
Superior frontal gyrus	6	L	−15, 28, 58	4.42	25	−10, 18, 60	5.20	79	−14, −4, 70	4.87	16			
		R				14, 30, 58	4.34	134						
	8	L	−24, 28, 56	5.26	1338	−22, 38, 48	6.88	24						
			−38, 18, 56	5.18	16									
		R				4, 30, 48	7.06	134						
						14, 34, 50	5.15	134						
	9	L	−14, 50, 36	5.23	1338									
	10	L	−16, 60, 18	4.58	69									
			−16, 54, 26	4.73	15									
		R	24, 58, 6	4.00	48	20, 66, 12	5.05	12						
														
**CINGULATE CORTEX**														
Anterior cingulate	32	L				−2, 50, 2	5.63	24				−12, 32, −10	4.69	22
		R				4, 34, 20	4.29	17				22, 40, 16	5.26	19
						5, 38, 15	4.51	16						
	24	L				−4, 30, 20	5.61	17						
						0, 16, 24	4.91	14						
Cingulate gyrus	32	L							−16, 24, 28	6.73	81	−20, 8, 42	4.03	17
	24	R							10, 4, 48	4.37	33			
		L										−20, −18, 46	4.15	52
	31	R										22, −50, 28	4.39	45
**TEMPORAL CORTEX**														
Middle temporal gyrus	21	L	−64, −54, −2	6.52	797									
		R	64, −58, 0	5.41	689									
			62, −2, −28	5.01	109									
	38	L	−38, 4, −40	3.93	43									
Superior temporal gyrus	22	L	−65, −55, 10	5.88	797	−60, −52, 10	5.25	53						
	38	L				−25, 14, −30	4.55	57						
Inferior temporal gyrus	37	R	56, −46, −22	4.56	689									
														
Insula	13	L										−30, −38, 14	4.74	101
**PARIETAL CORTEX**														
Precuneus	7	R							20, −55, 30	8.11	1216			
	7	L							−14, −58, 52	7.09	844			
									−12, −66, 30	5.44	75			
									−22, −68, 30	4.12	13			
	31	L										−20, −52, 38	3.79	30
Superior parietal lobule	7	R							26, −66, 58	9.35	1216			
		L							−24, −66, 48	7.47	844			
Postcentral gyrus	3	L							−40, −36, 52	5.35	201			
Inferior parietal lobule	40	R	68, −46, 24	5.09	689				42, −40, 56	4.83	19			
		L	−66, −30, 28	9.88	797									
			−52, −35, 40	4.55	27									
**CAUDATE**														
Body		L							−20, −24, 22	9.00	111	−4, 14, 10	5.19	13
		R										14, −18, 30	4.02	62
Tail		R							24, −32, 16	6.01	10	34, −46, 8	4.23	104
												32, −30, −2	3.81	12
**CEREBELLUM**														
Posterior lobe, tonsil		R	28, −52, −40	10.6	1267									
Posterior lobe, tuber		R	34, −70, −35	7.44	1267									
Posterior lobe, pyramis		R	14, −64, −38	6.65	1267									
Anterior lobe		R				24, −56, −38	6.74	18						

#### Areas in which the tiapride group showed reduced activation

**Figure 5:**
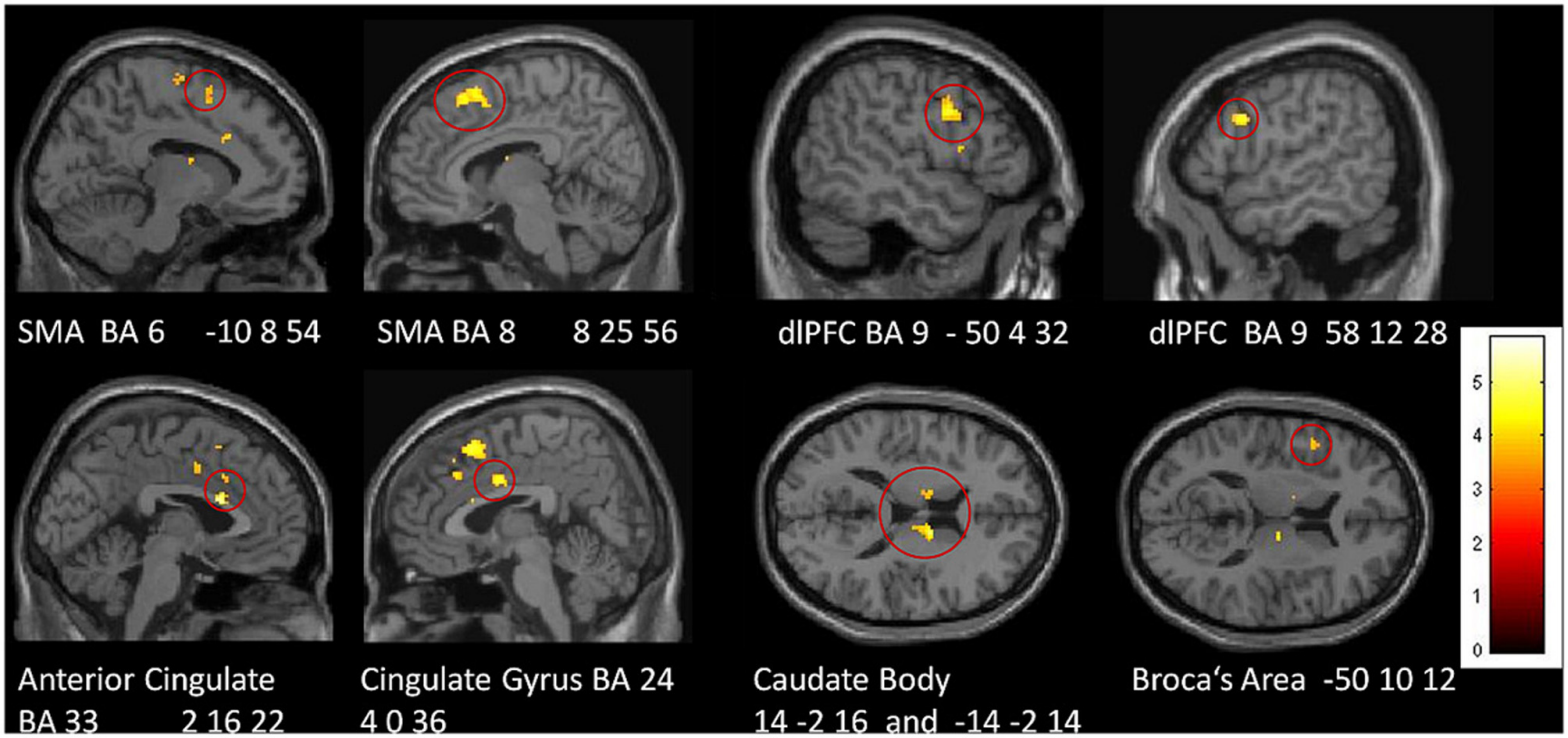
**Areas of reduced activation in the tiapride group, compared to the placebo group, in reproduction of the learned motor sequence (two-sample *t*-test, contrast placebo > tiapride reproduction > improvisation, thresholded at *p* < 0.001 uncorrected, minimum cluster size = 10 voxel)**.

In a direct comparison between groups, we identified those regions in which the tiapride group showed reduced activation compared to placebo (two-sample *t*-tests, placebo > tiapride for improvisation > reproduction and reproduction > improvisation, *p* < 0.001, height threshold 3.43, extent threshold *k* = 10). During reproduction, the tiapride group exhibited less activation in: bilateral anterior cingulate/cingulate gyrus (BA 24, 32, 33), bilateral supplementary motor area (BA 6, 8) dorsolateral PFC (dlPFC) (BA 9), bilateral caudate body, Broca's area (BA 44). In bilateral caudate tail, the tiapride group showed less activation than the placebo group during improvisation over the learned motor sequence (see Table 5 and Figure 5).

**Table 5:** **Reduced activation in the tiapride group compared to the placebo group (two-sample *t*-tests, contrast placebo > tiapride in improvisation > reproduction and reproduction > improvisation, *p* < 0.001, *k* = 10)**.

**Brain region**	**BA**		**Placebo > tiapride**					
			**Reproduction > improvisation**			**Improvisation > reproduction**		
			**MNI**	***t*-value**	**Voxel**	**MNI**	***t*-value**	**Voxel**
**SUPPLEMENTARY MOTOR AREA**								
Superior frontal gyrus	8	R	6, 18, 56	4.89	208			
	6	L	−10, 8, 54	3.74	17			
Medial frontal gyrus	8	R	8, 25, 56	4.34	208			
**DORSOLATERAL PFC**								
Inferior frontal gyrus	9	L	−50, 4, 32	4.74	125			
		R	58, 12, 28	4.39	42			
			42, 4, 28	3.85	13			
Superior frontal gyrus	6	L	−14, −8, 72	3.94	42			
Medial frontal gyrus	8	R	2, 30, 48	3.85	11			
	6	R	4, 28, 38	4.10	17			
Precentral gyrus (Broca's area)	44	L	−50, 10, 12	3.77	13			
**CINGULATE**								
Anterior cingulate	33	L	−2, 16, 22	5.82	119			
Cingulate gyrus	32	L	−2, 18, 36	3.77	119			
	24	R	4, 0, 36	4.33	61			
		L	−2, 2, 44	3.75	61			
**CAUDATE**								
Caudate body		R	14, −2, 16	4.97	50			
		L	−14, −2, 14	3.97	18			
Caudate tail		R				26, −44, 16	3.15	31
		L				−34, −42, 0	3.00	19

#### Correlations of performance and brain activation

During motor sequence reproduction, mean signal intensity in the following regions correlated positively with performance of the complete group: bilateral anterior cingulate, bilateral caudate body and left BA 6 (left anterior cingulate BA 32: Pearson's correlation coefficient *r* = 0.375; *p* = 0.027 two-tailed, right cingulate gyrus BA 24: *r* = 0.366; *p* = 0.030, left caudate body: *r* = 0.387; *p* = 0.023; right caudate body: *p* = 0.418 *r* = 0.015, left BA 6: *r* = 0.510; *p* = 0.003). Activation in Broca's area only showed a trend toward a significant correlation (*r* = 0.258; *p* = 0.097). In the supplementary motor area (SMA) regions and in dlPFC there were no significant correlations between performance and activation (SMA right *r* = 0.079; *p* = 0.348, left *r* = 0.211; *p* = 0.145; BA 9 right *r* = 0.221; *p* = 0.105, left *r* = 0.250; *p* = 0.105).

## Discussion

Corresponding to our hypotheses, our results demonstrate that DA-antagonism has opposing effects upon CE in motor cortex and upon motor sequence learning. Tiapride increased CE after motor training while simultaneously impairing motor learning and performance. In parallel to the behavioral effects, tiapride reduced activation in several regions of the brain network which processes motor sequence performance. In summary, our results do not support a concept of CE as a marker of motor learning, at least not for complex motor sequencing tasks.

### DA-antagonism enhances cortical excitability only if combined with motor training

Our findings indicate that the post-training increase in CE is associated with DA-antagonism, since a comparable change was found neither in the placebo group nor in the participants of a previous study using the same motor task in untreated participants (Lissek et al., 2013). This tiapride-induced enhancement reflected in significantly reduced inhibition and increased facilitation.

Notably, there was no immediate effect of the DA-antagonist upon baseline CE. Here, our results expand findings on the effects of dopaminergic manipulations upon CE which showed that in general, D2-agonists such as bromocriptine, cabergoline, and pergolide lower intracortical excitability, while D2-antagonists such as haloperidol increase it (Ziemann et al., 1997; Korchounov et al., 2007; Nitsche et al., 2010). However, these studies tested the impact of DA manipulation upon CE without an intervening learning element, therefore they are comparable to our comparison of baseline and pre-training CE. In contrast to the findings for haloperidol (Ziemann et al., 1997), tiapride did not significantly alter baseline CE. The differential effects may be due to dosage or divergent pharmacological profiles of these DA-antagonists (Kohler et al., 1984; Satoh et al., 1987). Other studies found enhanced CE after motor training alone (Abbruzzese et al., 1996; Muellbacher et al., 2001; Koeneke et al., 2006; Cirillo et al., 2010, 2011). In contrast to these findings, our results suggest that at least for complex motor sequence learning, training alone does not alter CE, since in the placebo group CE remained unchanged. Different experimental designs and methods of calculating CE might account for the diverging findings, which we discussed in detail in a previous report (Lissek et al., 2013).

Taken together, it is reasonable to assume that the CE increase was evoked predominantly by a combination of dopaminergic mechanisms and processes mediated by learning. Further research will be needed to investigate the precise conditions that can induce such a combined effect.

### DA-antagonism disturbs motor sequence learning and performance

DA-antagonism by tiapride was associated with disturbed motor sequence learning and performance. These results are in line with findings that relate administration of DA-agonists and antagonists, respectively, with enhancements or impairments in motor skill learning both in humans and animals (Floel et al., 2005; Molina-Luna et al., 2009). Complementing its behavioral effects, tiapride also had a prominent impact upon brain activation during motor performance. In particular during motor sequence reproduction, the tiapride group exhibited significantly lower activation in various brain regions related to working memory and motor sequence learning (bilateral SMA and dlPFC, ACC, cingulate gyrus, caudate body, and Broca's area), giving rise to the assumption that this reduced activation is associated with their learning deficit. Moreover, for bilateral caudate body, bilateral anterior cingulate and left BA 6, BOLD intensity correlated with the percentage of correct responses across groups, suggesting that the processing functions in these regions contribute specifically to successful performance in the motor task.

Tiapride has effects upon striatal D2 receptors (Dose and Lange, 2000), presumably causing the reduced activation in caudate body in our experimental group, which might be a key factor for their impairment. The correlation of caudate body activation with performance corresponds to findings that attribute to dorsal striatum/caudate an important role in executing learned motor programs (Dose and Lange, 2000) and in performance improvements during motor sequence learning (Albouy et al., 2012), with a more consistent performance being related to higher caudate activation. Antagonistic effects of tiapride have also been demonstrated for limbic structures (Scatton et al., 2001). In the present study, tiapride administration was associated with lower activation in limbic and frontal regions during motor performance, suggesting an inhibiting effect of the DA-antagonist which is consistent with effects of haloperidol and sulpiride upon prefrontal regions (Moghaddam and Bunney, 1990; Tzschentke, 2001). Anterior cingulate/cingulate gyrus activation in motor learning is associated with processing of sensory feedback (Jueptner et al., 1997) and with accurate temporal adjustments between hands in a bimanual motor task (Stephan et al., 1999). With temporal adjustment being a crucial requirement in our task, the reduced cingulate activation in the tiapride group might be connected to their inferior coping with the bimanual task.

Activation in dlPFC, SMA, and Broca's area showed no significant correlation with the performance level of participants. The SMA participates in learning of sequenced actions (Jenkins et al., 1994; Halsband and Lange, 2006) and in retrieval of already learned sequences (Grafton, 2009). SMA activation increased (Grafton et al., 2002; Daselaar et al., 2003) and correlated with explicit sequence learning in a serial reaction time task (Honda et al., 1998). DlPFC activity is presumably related to working memory recall of explicitly learned motor sequences (Grafton, 2009). Therefore, the placebo group's higher activation of these regions might be associated with their more successful retrieval of the learned motor sequences, or with the effort invested in retrieval, but not the success, given the lack of a significant correlation. Broca's area has been associated with motor sequence learning (Clerget et al., 2009, 2011, 2012, 2013), therefore its more prominent activation in the placebo group could be related to their superior learning.

While the tiapride group displayed reduced activation in several cortical regions outside motor cortex, their activation in motor cortex was higher than placebo. Motor cortex activation, however, was uncorrelated to CE and performance quality, except for a negative correlation across all subjects in a region in right BA 6 suggesting a relation between higher activation and inferior performance. However, the significance of this single evidence is questionable, since none of the other motor regions displayed such a correlation. Despite a lack of significant correlation between the two measures, the tiapride group's overall higher motor activation during task performance might be related to their higher CE measured post training, potentially indicating local plasticity processes restricted to the motor region that—due to task complexity and other brain regions involved—had no beneficial effects upon performance success.

### Altered improvisation behavior in tiapride participants

Improvisation performance in the tiapride group differs from that in the placebo group. However, their improvisation deficit, which manifests predominantly in less participants performing proper improvisations, probably results from inadequately acquired motor sequences. Therefore, it appears problematic to relate the impairment directly to the action of the DA-antagonist, despite the fact that cognitive flexibility has been shown to be associated with D2 receptor activity (Van Holstein et al., 2011; Stelzel et al., 2013) and thus may have been compromised by tiapride. A further hint that cognitive flexibility might have been impaired, though, is the observation that more tiapride than placebo participants with reproduction correctness above 50% tended to stick to the learned sequence instead of improvising over it.

### Increase in cortical excitability is not necessarily related to learning success

The present findings complement the results of a previous study (Lissek et al., 2013) in which we demonstrated in healthy participants that CE remained unchanged after learning and training of an identical motor sequence task. Moreover, the inverse relation of CE and learning performance found in the tiapride group corresponds to the findings of a study using the DA-agonist cabergoline, which for a simple motor practice task of thumb movements showed an increase in practice-dependent plasticity combined with a decrease in CE (Meintzschel and Ziemann, 2006). In the same study, the DA-antagonist Haloperidol decreased practice-dependent plasticity. Our results extend the findings of this study by demonstrating a similar dissociation of parameters for explicit learning of a complex motor sequence task. Overall, the findings suggest that an increase in CE is not necessarily related to learning success, but that successful learning can occur without a change in CE.

Interestingly, recent studies in transgenic mice found similar discrepancies between excitability/synaptic plasticity in hippocampus and performance in associative learning: increased excitability evoked by overexpression of TrkC receptors on the one hand and sustained enhancement of CREB (cAMP response-element binding protein) expression on the other did not produce a proportionate increase in learning a trace conditioning task or impaired performance of classical eyeblink conditioning, respectively (Sahún et al., 2007; Gruart et al., 2012). These are hints that also in other instances of learning and brain regions the link between measures of excitability and/or neuronal plasticity and performance parameters is not necessarily very close.

## Limitations

Considering some limitations of our study, we have to mention the uncorrected statistical thresholds we used for our imaging data, because the computed contrasts between Improvisation and Reproduction were very close in terms of processing requirements, i.e., the expected effects were rather small. Since uncorrected statistics were used, interpretation of imaging results should be done with caution.

Moreover, one might argue that the use of a circular coil for CE measurement does not extract reliable measures compared to a figure-of-eight coil, since one cannot make specific claims about a specific muscle change here. However, Ugawa et al. demonstrated comparable results between a figure-of-eight coil over left hand motor area and a circular coil over vertex for the determination of corticocortical facilitation and inhibition (Ugawa et al., 1994). In TMS studies in our department, the circular coil is used preferentially, since we observe stable and reproducible results; moreover, the positioning of this coil is less critical and therefore less error-prone (Schwenkreis et al., 2002, 2003, 2010).

## Conclusion

To the best of our knowledge, this study is the first to investigate the effects of dopaminergic antagonism upon complex motor learning and CE in a single setup. The results demonstrate contrary effects of D2/D3 blockade upon motor learning on the one hand and motor CE on the other: While motor sequence learning and performance proper were disturbed by the DA-antagonist, motor CE was found enhanced by the combination of task training and the DA-antagonist.

Our findings indicate that a simple straightforward relation between increased motor CE and higher learning success does not apply to every instance of a motor task. At least for complex motor sequence tasks that recruit a network of brain regions outside motor cortex, the effects appear to depend on various parameters, and CE in primary motor cortex is probably not the central determinant for learning success.

## Author contributions

Silke Lissek designed the study, acquired, analyzed, and interpreted the data, and drafted the paper. Guido S. Vallana analyzed data and drafted the paper. Lara Schlaffke and Melanie Lenz acquired data and revised the manuscript. Hubert R. Dinse and Martin Tegenthoff designed the study, interpreted data, and revised the manuscript. All authors approved the final version of the manuscript.

### Conflict of interest statement

The authors declare that the research was conducted in the absence of any commercial or financial relationships that could be construed as a potential conflict of interest.
